# Emissions of Volatile Organic Compounds from Brake Wear and Their Role in Ultrafine Particle Nucleation

**DOI:** 10.1021/acsestair.5c00070

**Published:** 2025-06-11

**Authors:** Olivier Durif, Lucas Bard, Karine Elihn, Barbara Nozière, Ulf Olofsson, Sarah S. Steimer

**Affiliations:** † Department of Chemistry, 7655 KTH, Royal Institute of Technology, Stockholm 10044, Sweden; ‡ Department of Machine Design, KTH, Royal Institute of Technology, Stockholm 10044, Sweden; ¶ Department of Environmental Science, Stockholm University, Stockholm 11418, Sweden; § Bolin Centre for Climate Research, Stockholm 11418, Sweden

**Keywords:** Brake wear, Ultra fine particle, FMPS, PTR-TOF-MS, NAO, LMCF, VOCs, Tribology

## Abstract

The emission of volatile organic compounds (VOCs) from brake wear is a relatively underexplored aspect of nonexhaust traffic emissions. We employed a proton-transfer-reaction time-of-flight mass spectrometer to investigate the real-time emissions of VOCs from two commercially prevalent brake materials: low-metallic copper-free (LMCF) and nonasbestos organic (NAO). Experiments were conducted using a pin-on-a-disc tribometer integrated with a fast-mobility particle sizer spectrometer. The results reveal that NAO brake pads emit higher concentrations of VOCs than LMCF pads. Over 200 different sum formulas were identified for both materials, and siloxanes were observed as characteristic of NAO emissions. The ultrafine particle formation was systematically preceded by an increase in gaseous emissions. This observation supports the thesis that ultrafine particle formation emitted by brake wear occurs through VOCs nucleation, addressing a significant gap in the literature.

## Graphical abstract

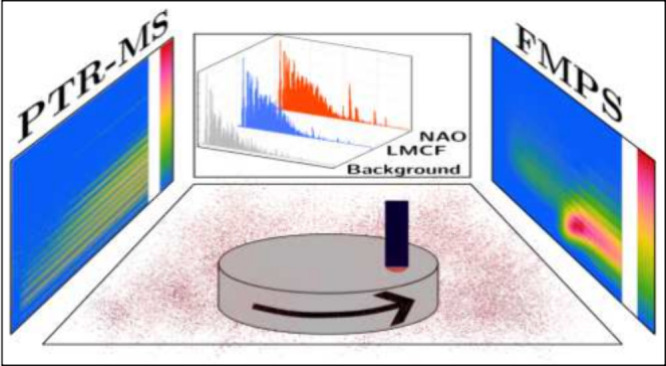

## Introduction

Today, due to notable reductions in exhaust emissions from fuel combustion, the contribution of nonexhaust sources to urban particulate matter now often equals or even exceeds contributions from exhaust in high-income countries.^1,2^

The braking process, crucial for vehicle safety, is one important source of nonexhaust emissions.^1^ While particles emitted from brake wear have been intensively studied over the last decades, gaseous emissions remain a relatively underexplored topic, and their composition, environmental importance, and impact on human health are not yet clear.

Brake pad formulations can contain a wide range of both organic and inorganic components. Possible organic components include phenolic resins used as binders, aramid fibers used for reinforcement, and fillers made from natural materials such as cork, rubber, and cashew dust.^3^ Friction during the braking process generates heat, which can give rise to temperatures up to 180 °C or even 600 °C during a hill descent.^4^ At these temperatures, the organic components can undergo pyrolysis or combustion, which might lead to the release of volatile organic compounds (VOCs). This has e.g. been shown for the pyrolysis of phenolic resins, usually starting at around 300–400 °C,^5–7^ with the products depending on both the temperature and the heating rate.^6^ However, even at lower temperatures, thermogravimetric and calorimetric analyses of brake pad samples showed the onset of a notable mass loss between 250 and 300 °C, attributed to the degradation of the binder.^8,9^ Further evidence of the volatilization of organic brake components during the braking process is that particles emitted from braking often have a lower carbon content than that of the original brake lining. In addition, this discrepancy increases with the severity of braking.^10–12^

Only a few studies have directly measured the VOCs and SVOCs (semivolatile organic compounds) emitted from braking. An earlier study concluded that hydrocarbon emissions can account for a significant amount of mass loss in tests conducted at higher temperatures.^13^ Two more recent studies have provided information about the chemical composition of these emissions. One study used Pyr/GC/MS (pyrolysis-gas chromatography/mass spectrometry), FTIR (Fourier-transform infrared spectroscopy), and carbon phase analysis to identify approximately one hundred VOCs and SVOCs released from a model low-metallic brake pad.^14^ The other study, using PTR-MS and AMS (aerosol mass spectrometer), also reported a similar number of VOCs, providing information on emissions from ceramic and semimetallic brake pads and noting similarities to biomass burning.^15^

The emission of organic gases from the friction interface has also been linked to the emission of nanoparticles from braking. Nanoparticle concentrations increase strongly once a critical temperature is exceeded.^16,17^ Previous studies have hypothesized that these nanoparticles are formed, once the pad reaches a critical temperature, from the gases emitted through vaporization at the friction interface as a consequence of the release of frictional heat.^9,18,19^ The degradation of the organic pad components has been suggested as a potential source of these gas emissions.^9,19^ Several studies have confirmed the semivolatile nature of the compounds involved in the formation of nanoparticles.^20,21^ The nanoparticles also have a high carbon content,^9,22^ which further points toward SVOCs as likely precursors. In addition, a previous study demonstrated that VOCs and SVOCs generated from brake pads can form secondary particulate matter (SPM) when exposed to oxidants in a potential aerosol mass (PAM) flow reactor.^23^ This earlier work established a causal link between gas-phase emissions from braking and the formation of the SPM upon photo-oxidation. However, it did not resolve the molecular identity of the emitted VOCs, nor did it investigate how the composition of gaseous emissions varied with brake pad material or mechanical stress.

In the present study, we used online mass spectrometry to explore the evolution of VOC emissions in real time, produced during simulated urban-driving braking conditions and for two commercially prevalent brake pad materials for light-duty vehicles. The present study reports the first results comparing the VOC emissions from a low-metallic copper-free (LMCF) material, typical for the European market, and those from a nonasbestos organic (NAO) material, commonly used in the United States.

## Materials and Methods

### Experimental Setup

To investigate the emissions of VOCs and PM from brake wear, a Pin-on-Disc (PoD) tribometer^24^ was integrated with a Fast Mobility Particle Sizer (FMPS) and a Proton-Transfer-Reaction Time-Of-Flight Mass Spectrometer (PTR-TOF-MS). This setup (Figure S1) allowed the simultaneous monitoring of VOCs and ultrafine particles emitted from the friction process.

The PoD tribometer used in this study had a horizontal rotating disc and a vertically dead-loaded pin. The applied mechanical stress was controlled by adjusting both the contact pressure and the rotational speed of the disc. The disc temperature was continuously monitored with an infrared thermometer.

### Brake Pad Materials

This work investigated two commercially available brake pad materials: Low-Metallic Cu-Free (LMCF) and Nonasbestos Organic (NAO). Both materials were tested with the same rotor material, made from gray cast iron. These two friction pairs are used in the same type of light duty vehicle sold on both the European (LMCF pad) and the US (NAO pad) market.

### Tribometer Test Conditions

The experiments were conducted with varying contact pressures and rotational speeds, mimicking a range of braking intensities to simulate real-world braking conditions. The contact pressure on the pin was adjusted to three distinct levels: 0.6, 0.9, and 1.1 MPa. Concurrently, the rotational velocity of the disk was varied from 1 to 4 m s^–1^. When scaled to the car, these sliding speeds correspond to mean vehicle speeds from 12 to 48 km h^–1^. These parameters reflect the typical stress conditions encountered during braking in urban driving environments.^25^ Each experiment was carried out for several minutes to up to 2 h, depending on the specific test. In contrast to previous studies using this setup,^25–32^ a zero air generator was used to supply a 15 L min^–1^ flow of clean air, rather than filtered room air, to improve the gas-phase background for the detection of VOCs. An overview of all conducted tests and the corresponding conditions is given in Tables S1 and S2.

### Volatile Organic Compounds and Particulate Matter Measurement

VOCs emitted during the braking process were analyzed in real-time using a Proton-Transfer-Reaction Time-Of-Flight Mass Spectrometry (PTR-TOF-MS, FUSION, Ionicon).^33^ Briefly, the chemical ionization of the sampled gas was performed by a proton-transfer reaction from the hydronium ion (H_3_O^+^). The mass spectrometer was set to detect ions up to a *m*/*z* of 700 focusing primarily on ions between *m*/*z* 10 and *m*/*z* 150 and with a resolution of approximately 8000.

Particulate matter sizes ranging from 5 to 550 nm were monitored along with the gaseous molecules using a Fast Mobility Particle Sizer (FMPS, Model 3091, TSI Inc.).

### Data Analysis

All data recorded in real-time during the experiments (mass spectra, particulate concentration, and temperature) were processed and analyzed using specifically developed in-house scripts. Data and processing scripts that allow for full reproduction of the results are provided in open access on Zenodo.^34^

## Results and Discussion

### NAO Emits More VOCs than LMCF

The results from this study demonstrate a significant difference in VOC emissions between the LMCF and NAO brake pad materials (Figure 1). The recorded mass spectra show hundreds of peaks identifiable by their mass-to-charge ratio, which corresponds to a specific elemental composition. However, many of these signals likely represent a sum formula from the contribution of serveral isomers and this complexity makes it unrealistic to report absolute concentrations of VOCs. The VOC concentrations measured in this study are therefore reported as ratios of the signal relative to the background measured before the tribometer was started.

**Figure 1:**
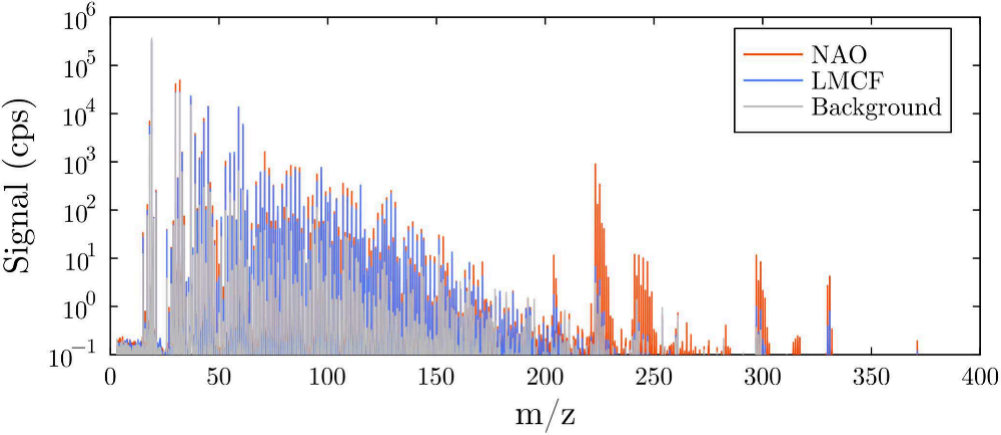
Mass spectra of NAO (red), LMCF (blue), and background (gray), highlighting the distinct emission profiles between the two materials under identical stress conditions. NAO emits siloxanes and overall more VOCs compared to LMCF. Spectra for both NAO and LMCF were averaged 19–20 min after the experiment began, with *c*_p_ = 1.1 MPa and *v* = 4 m s^–1^.

A general trend observed was that under identical mechanical stress conditions the NAO material consistently emitted higher concentrations of VOCs than the LMCF material (see Figure 1 and Figure S3). This difference is most evident in the emission of a group of peaks for *m*/*z* > 200, attributed to siloxane, which were detected in high intensities in the NAO material but were nearly absent in the LMCF material.

### Emitted VOCs Increase with Mechanical Stress

Both the contact pressure and the rotational speed of the disc were found to significantly influence the level of VOC emissions (Figure S2). Gas-phase emissions thus increased proportionally as the mechanical stress or as the disc temperature rose. These findings suggest that temperature is a reliable indicator for a given brake pad material to predict VOC emissions during brake wear. This correlation between brake temperature and VOC emissions was also recently pointed out by Perraud et al.^15^ Under the most severe testing conditions (1.1 MPa contact pressure, 4 m s^–1^ velocity), the total gas-phase signal for LMCF increased five times, while for NAO, it surged nine times relative to the baseline measurements (Figure 2). Although NAO emits overall more VOCs than LMCF in these extreme conditions, the disc temperature increased to only approximately 200 °C while it went up to 240 °C for LMCF. This difference in disc temperature is due to the difference in the friction coefficient between the NAO and LMCF pad. A NAO pad is a comfort pad material, with a lower friction coefficient between the pad and the rotor, than the LMCF pad, which is a performance pad material. Note that under these laboratory conditions the disc temperature was always below the maximum surface temperature to avoid excessive wear and problems with fading.^35^

**Figure 2:**
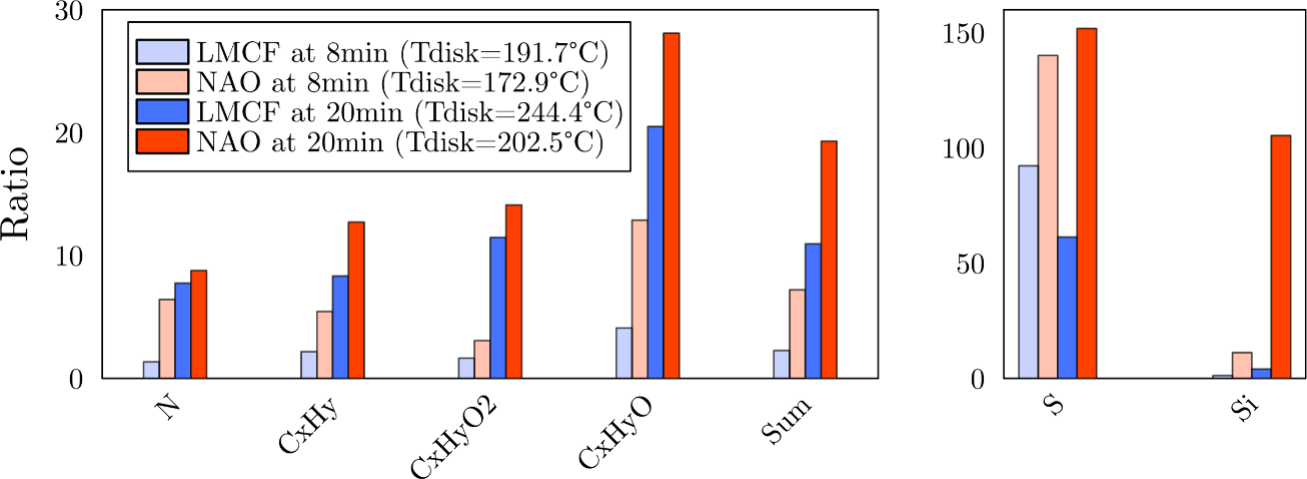
Signal for six different groups of molecules, N, S, Si, C_*x*_H_*y*_, C_*x*_H_*y*_O, and C_*x*_H_*y*_O_2_, containing atoms, plus the total sum signal. Reported relative to their background level and compared between NAO and LMCF. Tribometer conditions were *c*_p_ = 1.1 MPa and *v* = 4 m s^–1^. Two wear levels compared: after 8 min and after 20 min of friction. Two panels are displayed for scaling purposes.

### VOCs Composition and Potential Tracers

The identification of individual VOCs was difficult due to the multitude of compounds present, as shown in the mass spectrum (Figure 1). Each peak can correspond to several isomers but also, for a certain fraction, to a potential overlap with isotopes. To enable the attribution of a specific molecular formula to a specific peak, we developed an automated peak identification method. This method, further explained in the Supporting Information (SI), attributes a sum formula and signal level to each of the observed peaks. The corresponding list is hereafter referred to as “peak table”. When applied to the most extreme experimental conditions, the peak table includes over 210 molecular formulas. Thus, the wear from both pads yielded a minimum of 210 different VOCs, although most sum formulas likely represent multiple isomers. Peak tables obtained under these extreme conditions are reported in the SI for both the LMCF and NAO materials, while data for each experimental condition are provided in CSV files on Zenodo.

The molecular formulas were classified into six different groups based on their elemental composition: those containing N, S, or Si atoms and hydrocarbons and carbon compounds with 1 or 2 oxygen atoms. The total signal ratios for the different groups, relative to their signal in background air, are presented in Figure 2 for two identical mechanical stress conditions, comparing NAO and LMCF. Overall, the range of observed VOCs is similar between the NAO and LMCF pads, with the noteworthy exception of siloxanes. Such similarity in overall VOC composition was also observed by Perraud et al. between ceramic and semimetallic brakes.^15^ One novel insight from our results is that sulfur-containing compounds appear to be characteristic of brake wear, with a ratio up to 150 times relative to the background. However, the absolute signal of this group of compounds is fairly low, which might limit their utility as tracers in environments where the detection of such traces is challenging. For NAO, the presence of silicon-based compounds becomes particularly prominent under high mechanical stress conditions, making it a distinguishing characteristic of this material and a potential tracer.

Most of the organic compounds attributed to the proposed sum formulas were those classically detected by PTR-MS^36^ such as ketones, alkenes, alcohols, aldehydes, and carboxylic acids. Detected ketones (R–C­(=O)–R′) included propanone (C_3_H_6_O), butanone (C_4_H_8_O), or potentially molecules like acetophenone (C_8_H_10_O) and phenylethanone (C_8_H_10_O). Alkenes (C_*n*_H_2*n*_) were observed up to *n* = 7 but larger alkenes such as C_11_H_22_ were also monitored. However, they were only reported up to *n* = 4 in the peak table because for the ones above that number their low signal is below the threshold defined in our method. Alcohols (R–OH) identified based on the atomic composition were monohydric as methanol (CH_3_OH), ethanol (C_2_H_5_OH), propanol (C_3_H_7_OH), butanol (C_4_H_9_OH), pentanol (C_5_H_11_OH), or hexanol (C_6_H_13_OH) or polyhydric as ethanediol (C_4_H_4_(OH)_2_), propanediol (C_3_H_5_(OH)_3_), butanediol (C_4_H_8_(OH)_2_), or even propanetriol (C_3_H_5_(OH)_3_) and butanetriol (C_4_H_6_(OH)_3_). Aldehydes (R–CH=O) were observed from 1 up to at least 10 carbon atoms $\left({\left(C_{n}H_{2n}O\right)}_{1\leq n\leq 10}\right)$ . Aromatic aldehydes were also likely observed, such as benzaldehyde (C_7_H_6_O). However, these chemical formulas might also represent other isomers, such as ketones. Some of the observed peaks corresponding to amines were detected, although with a low signal level, such as dimethylamine (C_2_H_7_NH^+^) or triethylamine (C_3_H_9_NH^+^). However, these peaks could also correspond to adducts of ammonium ions with alkenes that might potentially occur during chemical ionization. Carboxylic acids (R–C­(=O)–OH) or esters (R–C­(=O)–O–R′) of the general formula C_*n*_H_2*n*_O_2_ were too numerous to be listed. These compounds were present for *n* = 14 minimum. Detected aromatic hydrocarbons included toluene (C_7_H_8_), benzene (C_6_H_6_), ethylbenzene, xylenes (C_8_H_10_), and phenol (C_6_H_6_O). This is consistent with the emission of BTEX compounds (Benzene, Toluene, Ethylbenzene, and Xylenes) previously reported from simulated wear of a low-metallic pad.^14^ Another study found that aromatics represented respectively 29% and 12% of the VOCs emitted from ceramic and semimetallic brakes, benzene being the most prominent.^15^ Particularly under fade conditions, when the brakes lose their effectiveness due to overheating, BTEX compounds could be degassed from the original brake linings.^14^ However, as also observed in moderate stress conditions, they might potentially be formed, for instance, during the braking process via thermal degradation of the phenolic resin binders, as previous studies have demonstrated pyrolytic formation of benzene, toluene, and xylene from different phenolic resins.^6,37,38^

Among other noticeable compounds were acetonitrile (C_2_H_3_N) and also C_3_H_4_O and C_5_H_4_O_2_, observed in a large amount. Some of these compounds could come from the solvents used during the pad’s manufacture. Others detected ions were C_2_H_2_O^+^ and C_3_H_3_^+^, which are unstable and thus likely fragments produced from larger neutral parents during ionization. Last but not least, sulfide compounds were also observed, and among them, carbon disulfide, CS_2_, is the molecule with the highest intensity relative to the background. This molecule is, therefore, a prime candidate as a tracer of the effect of brake wear for field measurements.

The major difference between the spectra for LMCF and NAO was observed in the mass range *m*/*z* > 200 (see Figure 1), where the signals for the NAO material were up to 2 orders of magnitude larger than for the LMCF material. These peaks have been attributed to siloxanes based on their mass and were 100 times more intense at high friction stress than in the background experiment (see Figure 2). Such groups of peaks have been observed independently of this study with the same PTR-MS, originating from glass dust. This suggests that they may be emitted by any glass fiber incorporated during the manufacturing process.

### Ultrafine Particle Formation

The formation of ultrafine particles (<100 nm) was observed during braking under conditions of high mechanical stress and elevated temperatures. Above a critical threshold temperature, particles displayed a “banana plot” pattern (see Figure 3 and Figure S3), typical of particle nucleation followed by growth via condensation and coagulation processes.^39^ This pattern was systematically observed once the disc temperature exceeded 120 °C, the most frequent temperature onset being around 150 °C. This critical temperature variation from one experiment to another (see Figure S4) might be due to inhomogeneities in the brake pad materials. The emission factors of the NAO pad material, in particular, have been reported to display a large variability.^40^ However, they may also reflect the complexities of the nucleation and growth process, which depends on many factors, including the presence of other particles as a competing condensation sink and the availability of easily nucleating inorganic gases such as sulfuric acid. Most importantly, a distinct rise in gas-phase emissions, peaking between 130 and 160 °C, consistently coincided with, and often preceded by 10–20 °C, the appearance of ultrafine particles (see Figure S4). Organic gas emission and the apparition of ultrafine particles were recently reported to correlate in the case of ceramic and semimetallic brakes.^15^ But, the present study is the first to establish that the increase in gas phase emissions actually precedes the formation of the ultrafine particles. This trend is illustrated in Figure 3, which shows the temperature relationship between gas emissions and particle formation for both LMCF and NAO materials at *c*_p_ = 1.1 MPa and *v* = 3 m s^–1^. This surge in VOC concentrations shortly before particle formation further supports the hypothesis that gaseous brake wear emissions are involved in the formation of ultrafine particles. It should be noted that most of the organic compounds observed with PTR-MS have a high volatility and therefore are not those directly involved in the particle formation. Semivolatile and low-volatility organic compounds, serving as a key step linking gas-phase emissions to ultrafine particle formation, will need to be investigated in future work.

**Figure 3:**
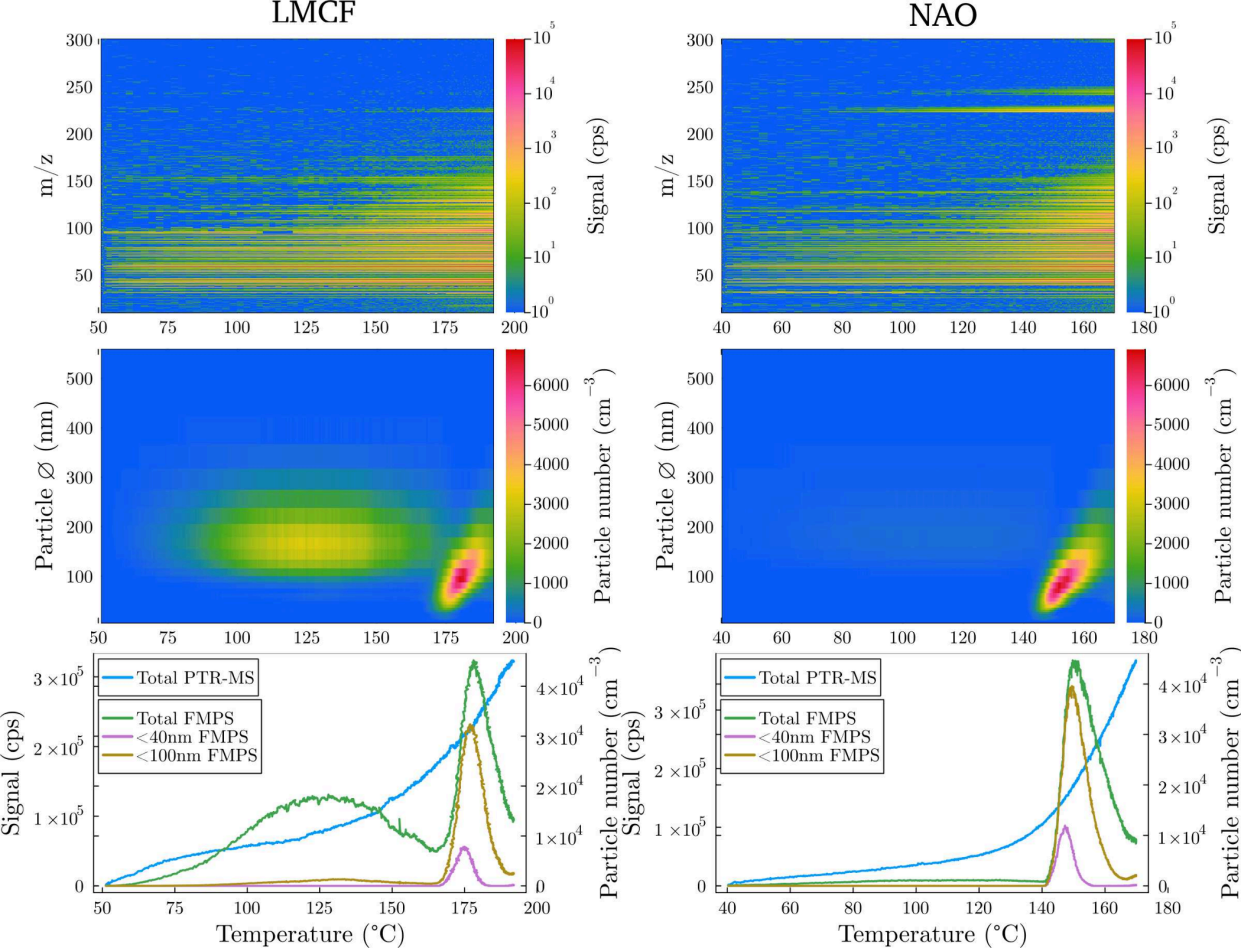
Comparison of LMCF (left column) and NAO (right column) under conditions *c*_p_ = 1.1 MPa and *v* = 3 m s^–1^, over a 20 min experiment following the temperature. Top row: gas-phase emission signals measured with PTR-TOF-MS. Middle row: particle signals measured with FMPS. Bottom row: summed signals. The temperature increases with time.

However, previous studies have suggested that the degradation of organic materials in brake pads may contribute to semivolatile and low-volatility organic compound formation.^9,18,19,41^ These studies compared the ultrafine and coarse fractions of particles generated from an iron braking disc paired with a LMCF material. The reported elemental and phase analyses identified two main compositional trends: first, a higher degree of iron oxidation, when moving toward the ultrafine fractions, and second, a higher concentration of carbon in the ultrafine particulates. Such an increase in the carbonaceous fraction for ultrafine particles has been observed previously^22^ and would be consistent with volatilized organics contributing strongly to the formation of these particles.

At temperatures under which ultrafine particle growth occurs, particulate matter emissions were frequently manifest as distinct spots on particle size distribution heatmaps. This phenomenon is evident in Figure 3, where such spots are observed for LMCF below 170 °C but in this example are absent for NAO. These observations are interpreted as particle emissions resulting from the abrasion of a thin surface layer on the pin during the friction process. This surface layer could be formed and regenerated through exposure to the atmosphere, primarily consisting of water and oxides that adhere to the surface of the metallic disc. However, the maximum intensity of these spots is generally lower compared to that when ultrafine particles are formed through nucleation (see Figure S3).

## Environmental Implications

The NAO brake pads, which are often marketed as environmentally friendly brake materials and less hazardous due to lower particle emissions,^16,21^ were found in this work to emit more gaseous pollutants compared to the LMCF. Many of the volatile compounds attributed to the observed peaks are known to be toxic for human health, which raises doubts on the actual benefits of these materials. In addition, the emissions occurred at conditions corresponding to mean vehicle speeds from 12 to 48 km h^–1^, where the disc temperature is well below 300 °C, which is contradictory to the common perception that gaseous emissions are only released at fading conditions. However, since the emission factors for VOCs from brake wear are still difficult to evaluate, the overall importance of the VOC emissions compared to those of the particles have not been studied yet. The determination of the emission factors of VOCs from brake wear is therefore an important subject for future studies.

The involvement of emitted gases in ultrafine particle formation through particle nucleation and growth has two important implications. On the one hand, it means that reducing gaseous emissions from brake pads could potentially mitigate the formation of ultrafine particles. On the other hand, it means that previously measured emission factors for ultrafine particles may not accurately reflect real-world conditions due to the complex interplay of gas-phase concentrations, condensation sink, and other factors. Therefore, further work will be necessary to validate these laboratory observations under more realistic braking conditions by using dynamometers or vehicle tests.

## Supplementary Material



## Data Availability

The raw data that support the findings of this article along with the data processing tools are openly available in Zenodo at https://doi.org/10.5281/zenodo.13869976, reference number 13869976.

## Notes

Detailed methods and materials, plus a description of the numerical analysis (PDF)
