# Supplementary material for: Emissions of Volatile Organic Compounds from Brake Wear and Their Role in Ultrafine Particle Nucleation
Source: ACS EST Air. 2025 Jun 11;2(7):1308–14. doi: 10.1021/acsestair.5c00070 (PMC12261270; doi:10.1021/acsestair.5c00070)
Supplement: Supplementary file 1 [file ea5c00070_si_001.pdf]

Supporting Information: Emissions of Volatile Organic Compounds from Brake Wear and Their Role in Ultrafine Particle Nucleation

Olivier Durif,[*,†] Lucas Bard,[‡] Karine Elihn,[¶] Barbara Nozière,[†] Ulf Olofsson,[‡] and Sarah S. Steimer[*,¶,§]

†*Department of Chemistry, KTH, Royal Institute of Technology, Stockholm, 10044, Sweden*

‡*Department of Machine Design, KTH Royal Institute of Technology, Stockholm, 10044, Sweden*

¶*Department of Environmental Science, Stockholm University, Stockholm, 11418, Sweden*

§*Bolin Centre for Climate Research, Stockholm, 11418, Sweden*

E-mail: durif@kth.se; sarah.steimer@aces.su.se

1 Experimental Setup

1.1 Proton-Transfer-Reaction Time-Of-Flight Mass Spectrometry

The chemical ionization is performed by proton transfer of neutral molecules, M, from the hydronium ion (H_3O^+) following this simple reaction:

$$M + H_3O^+ \longrightarrow MH^+ + H_2O.$$

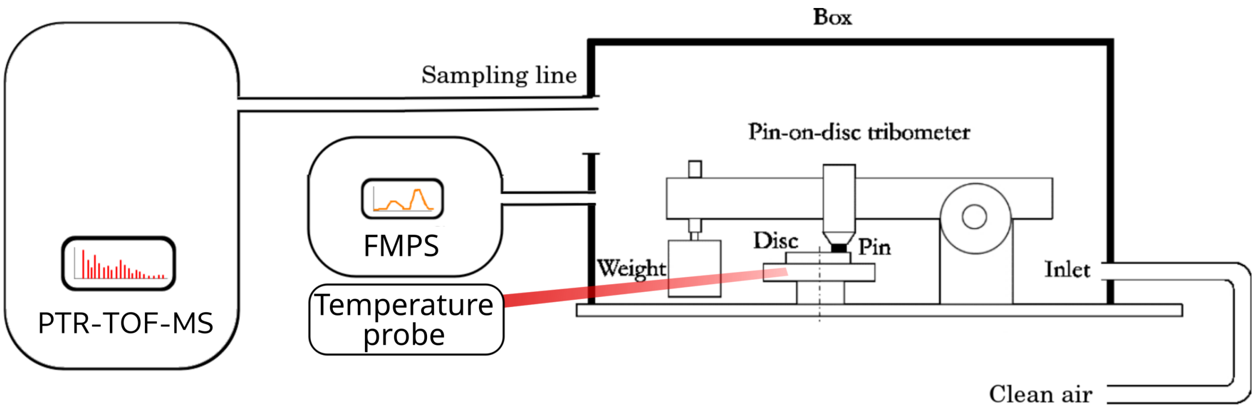

Figure S1: Experimental setup coupling a PoD tribometer with a PTR-TOF-MS, a FMPS, and an infrared thermometer.
PoD: Pin-on-Disk, PTR-TOF-MS: Proton-Transfer-Reaction Time-Of-Flight Mass Spectrometer, FMPS: Fast Mobility Particle Sizer.

Protonated molecules, MH^+, are then detected. But before, hydronium ions are produced in a discharge source and then merged with the flow sample from the tribometer in a drift tube of about 20 cm where the proton transfer reaction occurs at a few millibars of pressure. The resulting ions then pass through a hexapole and an electrostatic lens system before reaching the extraction zone of the time-of-flight (TOF). This TOF includes a reflectron and the mass resolution is about 8000. For this study, the ions are collected up to m/z of 700, using microchannel plates, and the signal is digitized by a Digital Signal Averager (ADQ7DC, Teledyne). The spectrometer configuration favored the detection of the lightest molecules ($m/z < 150$) and the sensitivity for molecules above 250 amu was low, making them nearly undetectable in trace amounts.

This spectrometer has been used in previous chemical kinetics studies.[S1,S2]

1.2 Flow Sampling

A Zero Air Generator (Sabio, model 1001) was utilized to supply a 15 L min^{-1} flow of dry, clean air into the tribometer. The sampling line connecting the tribometer and mass spectrometer consisted of a 2-meter-long, 1/4-inch PFA flexible tube. The gas flow rate sampled by the PTR-TOF-MS was 400 sccm. This corresponds to a transit time in the sampling

line of approximately 3 to 4 s. A disposable filter (Balston 9933-05-BQ) was installed at the entrance of the sampling line to remove particles (99.99 % at 0.01 µm), thus minimizing contamination of the mass spectrometer.

The air temperature inside the tribometer was kept constant for all experiments at 26.5(15) °C.

2 Experiments List

Different sets of experiments were conducted for the LMCF (table S1) and NAO (table S2) materials. Experimental conditions were varied with a contact pressure of 0.6 to 1.1 MPa and a disk velocity of 1 to 4 $m\,s^{-1}$. The acquisition time was between a few minutes to 2 hours depending on the trials.

Table S1: Low Metallic Copper-Free (LMCF) experiments list.

**Test №**	**Velocity (m/s)**	**Cp (MPa)**	**Test time (min)**	**Max. Disk Temp. (°C)**
2.1	1	0.6	15	-
2.2	2.5	0.6	15	-
2.2	4	0.6	120	-
3.1	2	0.6	11	83.0
3.2	2	0.6	16	-
3.3	2	0.6	16	87.4
3.4	2	0.6	10	78.7
3.5	2	0.6	11	86.4
3.6	2	0.6	8	85.8
3.7	2	0.6	6	81.9
3.8	2	0.6	6	82.5
3.9	2	0.6	6	82.4
4.1	4	1.1	20	234.3
5.1	1	1.1	20	72.9
5.2	2	1.1	20	147.5
5.3	3	1.1	20	202.7
6.1	4	0.6	120	164.6
6.2	4	0.6	120	182.9
7.1	4	0.6	120	143.4
9.1	2	0.9	20	101.3
9.2	3	0.9	20	153.1
9.3	4	0.9	20	189.2
9.4	4	0.9	20	196.7
10.1	3	1.1	20	195.1
10.2	4	1.1	20	247.0

Table S2: Non-Asbestos Organic (NAO) experiments list.

Test №	Velocity (m/s)	Cp (MPa)	Test time (min)	Max. Disk Temp. (°C)
11.1	1	0.6	20	49.4
11.2	2	0.6	20	92.4
11.3	3	0.6	20	125.0
11.4	4	0.6	20	145.7
12.1	2	0.9	20	135.8
12.2	3	0.9	20	169.9
12.3	4	0.9	20	196.5
13.1	1	1.1	20	82.6
13.2	2	1.1	20	134.1
13.3	3	1.1	20	172.0
13.4	4	1.1	20	211.3
14.1	2	0.6	6	76.3
14.2	4	0.6	6	100.5
14.3	3	0.6	11	102.7
14.4	4	0.6	8	111.7
15.1	4	0.6	120	132.2
15.2	4	0.6	120	152.0
16.1	4	0.6	120	165.4
16.2	4	0.6	120	159.4
17.1	3	0.9	20	145.4
17.2	3.5	0.9	20	171.5
17.3	4	0.9	20	185.9
18.1	3	1.1	20	183.6
18.2	3.5	1.1	20	196.9
18.3	4	1.1	20	214.6
19.1	0	0.9	65	-
19.2	4	0.9	65	180.9
19.3	4	0.9	65	187.7

3 Signal as Function of the Mechanical Stress

The signal for gaseous emission, particle formation, and disk temperature obtained as a function of the contact pressure and the disk velocity is represented as heatmaps below. PTR-MS heatmaps are constructed by always selecting data at the same experimental time (15 minutes). However, for the FMPS and temperature heamaps, the maximum signal is selected.

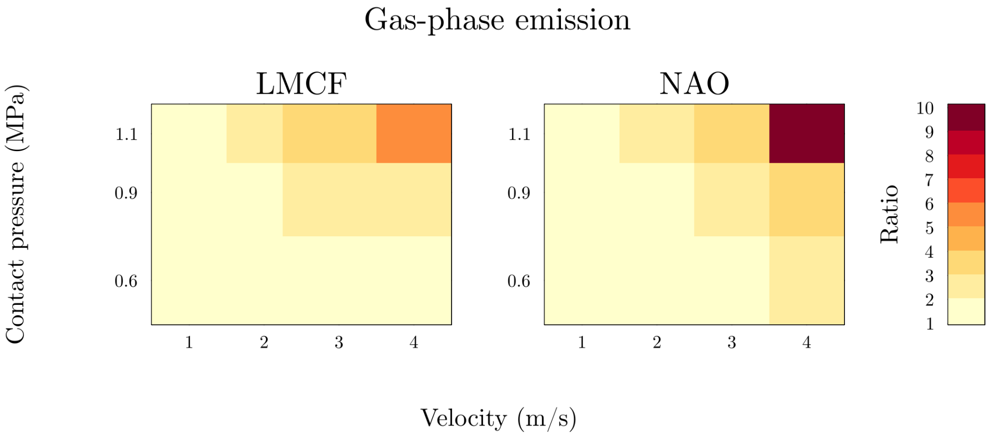

(a) VOCs signal is computed relative to the background. Gas emission intensity is correlated with the mechanical stress and than NAO emits more VOCs compared to LMCF.

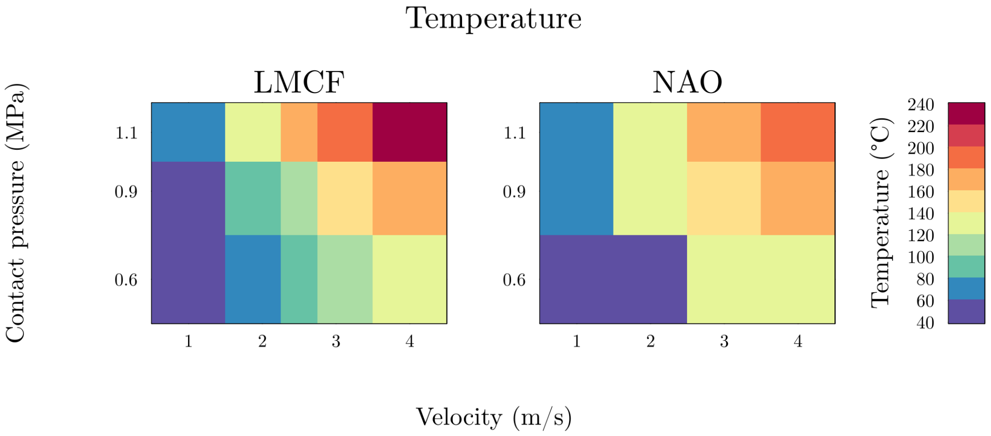

(b) Temperature warm-up when the mechanical stress is increased.

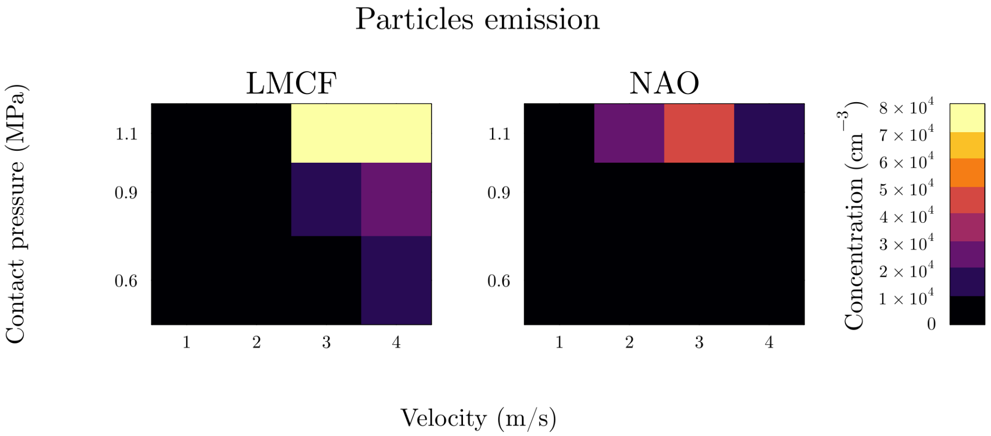

(c) Particles were abruptly formed in relatively high concentrations above a critical temperature.

Figure S2: Comparison of gas emissions, particles emission and temperature between LMCF (left) and NAO (right) materials under varying contact pressure (0.6,0.9 and 1.1 MPa) and disk rotation speed (1, 2, 3 and 4 $m\,s^{-1}$), measured consistently 900 s after tribometer activation.

3.1 Heatmaps Construction

Heatmaps are produced by summing the signal of various experimental conditions, hereafter refer by $P(c_p, v)$, where c_p is a given contact pressure and v is a given disk velocity. Below tables S3 and S4 provide a schematic of the different tests used to produce these heatmaps. For some missing experimental conditions, the corresponding data point is linearly interpolated based on the two nearest neighbors.

3.1.1 Heatmaps Construction for LMCF

Table S3: Tests used to construct heatmaps for the LMCF material. c_p is the contact pressure (MPa), v the disk velocity ($\mathrm{m\,s^{-1}}$).

5.1<br>$cp = 1.1, v = 1$	5.2<br>$cp = 1.1, v = 2$	$cp = 1.1, v = 2.5$	5.3<br>$cp = 1.1, v = 3$	10.2<br>$cp = 1.1, v = 4$
$cp = 0.9, v = 1$	9.1<br>$cp = 0.9, v = 2$	$cp = 0.9, v = 2.5$	9.2<br>$cp = 0.9, v = 3$	9.3<br>$cp = 0.9, v = 4$
2.1<br>$cp = 0.6, v = 1$	$cp = 0.6, v = 2$	2.2<br>$cp = 0.6, v = 2.5$	$cp = 0.6, v = 3$	6.2<br>$cp = 0.6, v = 4$

Missing data point, $P(0.6, 2)$, $P(0.6, 3)$, $P(0.9, 1)$, $P(0.9, 2.5)$, $P(1.1, 2.5)$ are interpolated as:

$$
\begin{aligned}
P(0.6, 2) &= \frac{1}{5}\left(2P(0.6, 1) + 3P(1.1, 1)\right), \\
P(0.9, 1) &= \frac{1}{5}\left(2P(0.6, 1) + 3P(1.1, 1)\right), \\
P(0.6, 3) &= \frac{1}{3}\left(2P(0.6, 2.5) + P(0.6, 4)\right), \\
P(1.1, 2.5) &= \frac{1}{2}\left(P(1.1, 2) + P(1.1, 3)\right), \\
P(0.9, 2.5) &= \frac{1}{2}\left(P(0.9, 2) + P(0.9, 3)\right).
\end{aligned}
$$

Note that no FMPS data was available for tests 2.1 and 2.2, thus the FMPS heatmap is reduced on 4 columns ($v = 1, 2, 3, 4\,\mathrm{m\,s^{-1}}$; no point at $2.5\,\mathrm{m\,s^{-1}}$).

3.1.2 Heapmaps Construction for NAO

Table S4: Tests used to construct heatmaps for the NAO material. c_p is the contact pressure (MPa), v the disk velocity ($\mathrm{m\,s^{-1}}$).

13.1<br>$cp = 3, v = 1$	13.2<br>$cp = 3, v = 2$	13.3<br>$cp = 3, v = 3$	13.4<br>$cp = 3, v = 4$
$cp = 2, v = 1$	12.1<br>$cp = 2, v = 2$	17.1<br>$cp = 2, v = 3$	17.3<br>$cp = 2, v = 4$
11.1<br>$cp = 1, v = 1$	11.2<br>$cp = 1, v = 2$	11.3<br>$cp = 1, v = 3$	11.4<br>$cp = 1, v = 4$

The missing data point, $P(2, 1)$, is interpolated as:

$$P(2, 1) = \frac{1}{5} \left(2P(1, 1) + 3P(3, 1)\right).$$

4 Time-resolved Data

$c_p = 0.6\,\mathrm{MPa}, v = 4\,\mathrm{m\,s^{-1}}$

(a) test 6.2 (LMCF)

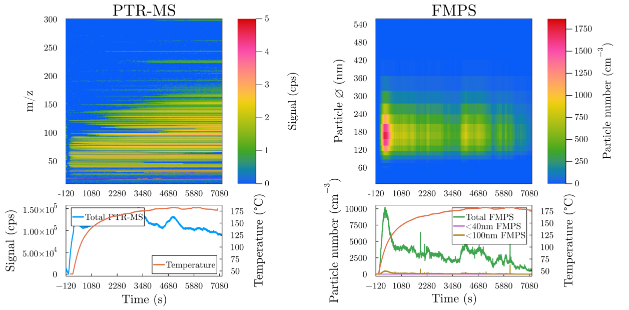

(b) test 15.1 (NAO)

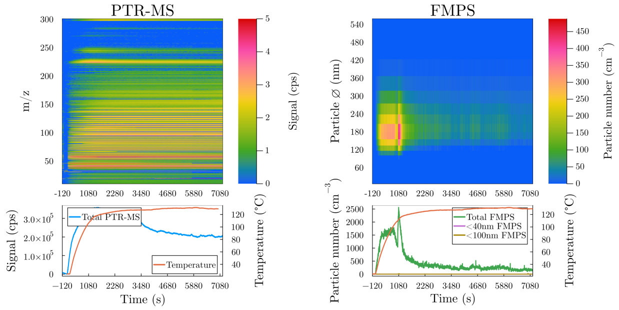

(c) test 7.1 (LMCF)

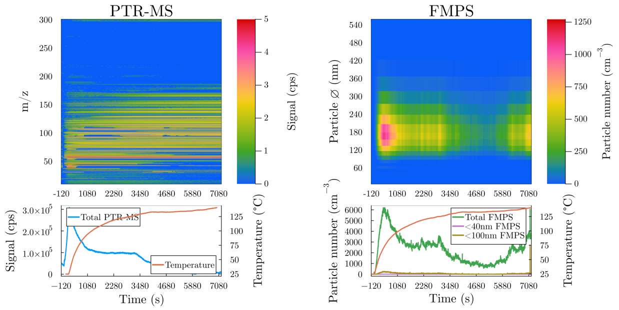

(d) test 15.2 (NAO)

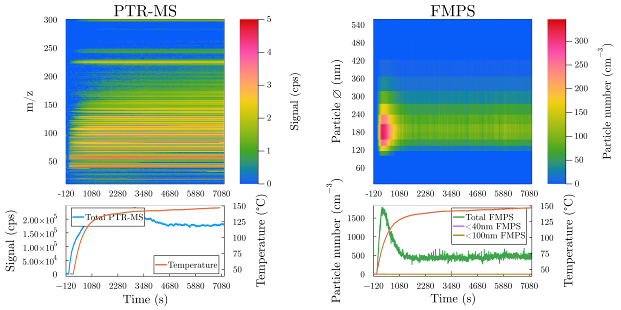

$c_p = 0.9\,\mathrm{MPa}, v = 2\,\mathrm{m\,s^{-1}}$

(e) test 9.1 (LMCF)

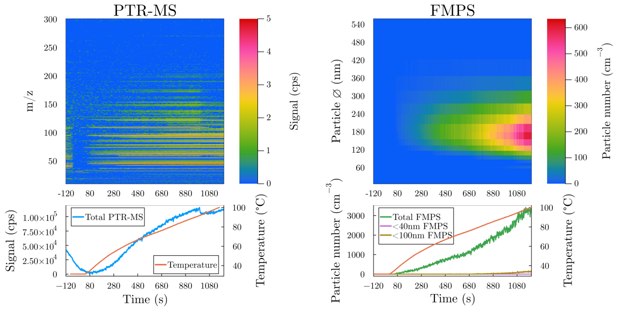

(f) test 12.1 (LMCF)

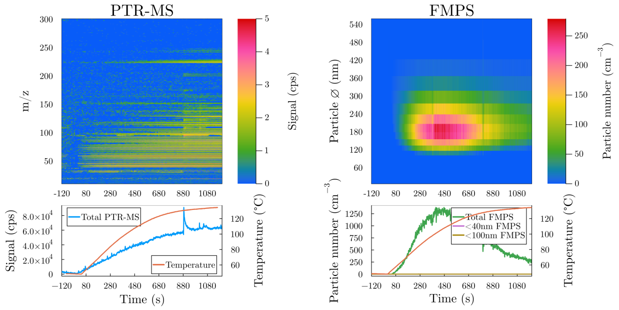

(g) $c_p = 0.9\,\mathrm{MPa}, v = 3\,\mathrm{m\,s^{-1}}$

(h) test 9.2 (LMCF)

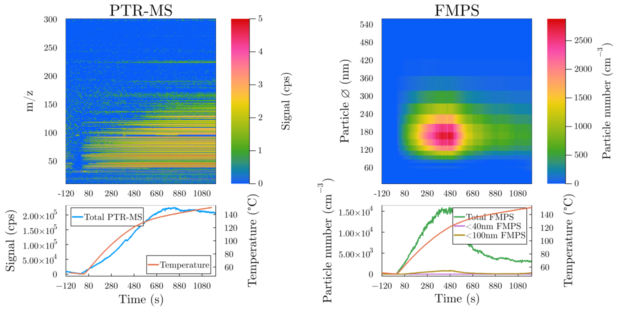

(i) test 12.2 (NAO)

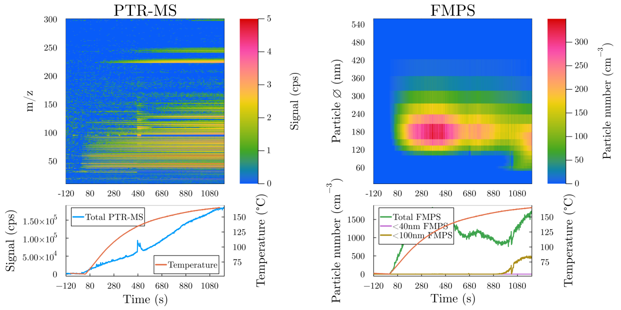

$c_p = 0.9\,\text{MPa}, v = 4\,\text{m}\,\text{s}^{-1}$

(j) test 9.3 (LMCF)

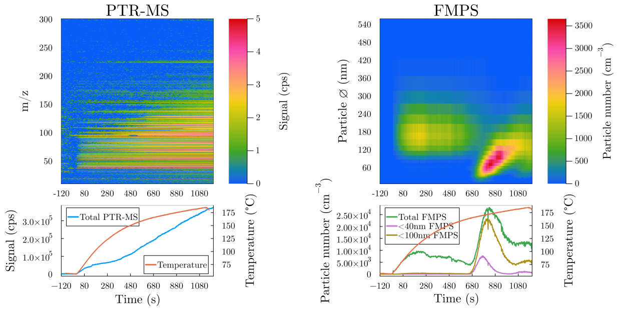

(k) test 12.3 (NAO)

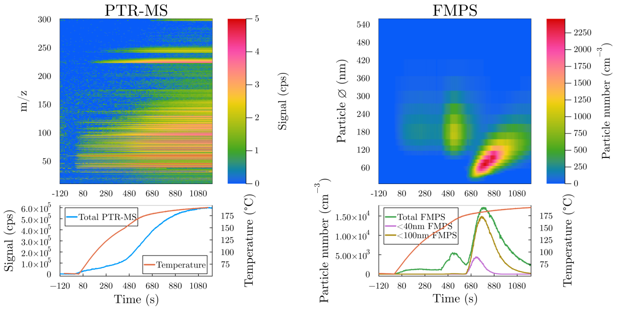

$c_p = 0.9\,\text{MPa}, v = 4\,\text{m}\,\text{s}^{-1}$

(l) test 17.1 (NAO)

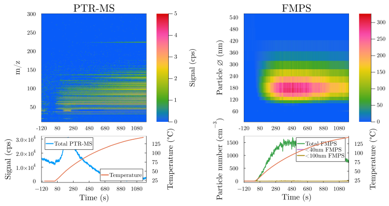

(m) test 17.2 (NAO)

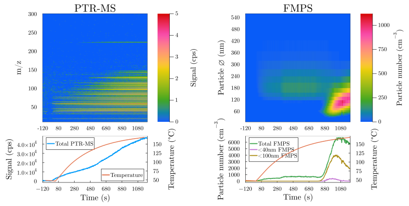

(n) test 17.3 (NAO)

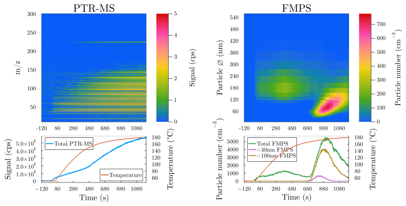

$c_p = 1.1\,\text{MPa}, v = 1\,\text{m}\,\text{s}^{-1}$

(o) test 5.1 (LMCF)

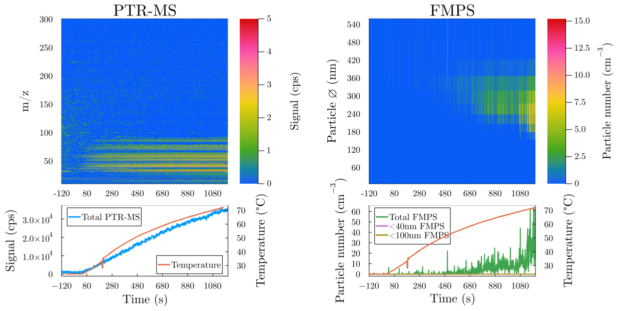

(p) test 13.1 (NAO)

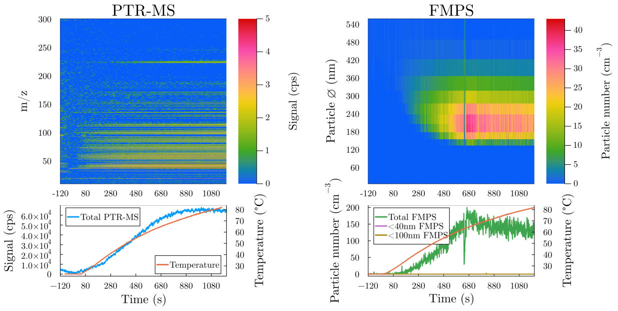

$c_p = 1.1\,\text{MPa}, v = 2\,\text{m}\,\text{s}^{-1}$

(q) test 5.2 (LMCF)

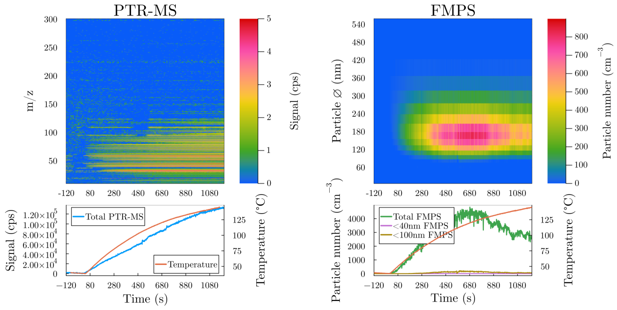

(r) test 13.2 (NAO)

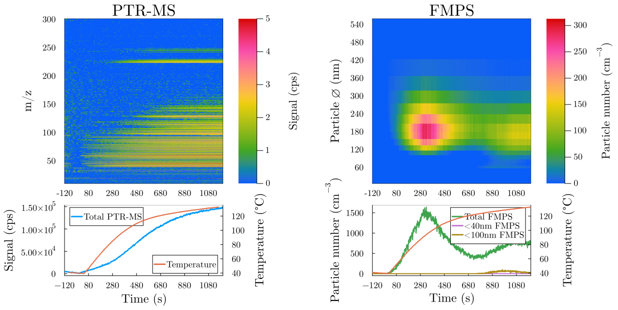

$c_p = 1.1\,\mathrm{MPa}, v = 3\,\mathrm{m\,s^{-1}}$

(s) test 10.1 (LMCF) (t) test 13.3 (NAO)

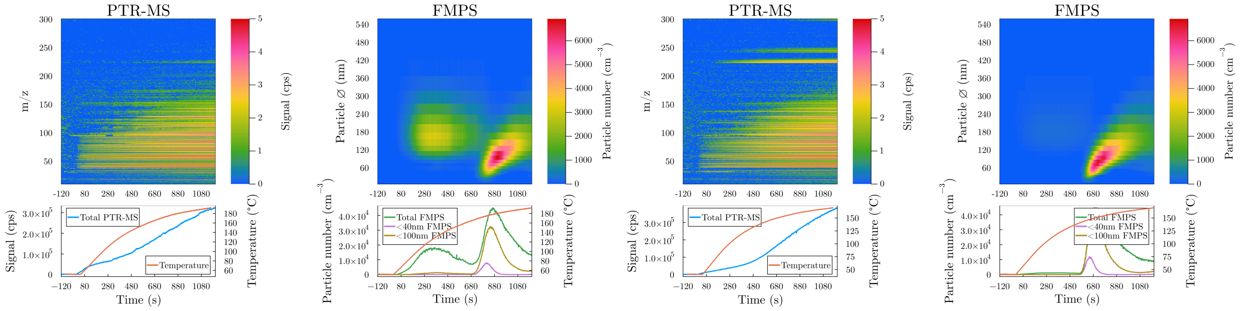

$c_p = 1.1\,\mathrm{MPa}, v = 4\,\mathrm{m\,s^{-1}}$

(u) test 10.2 (LMCF) (v) test 13.4 (NAO)

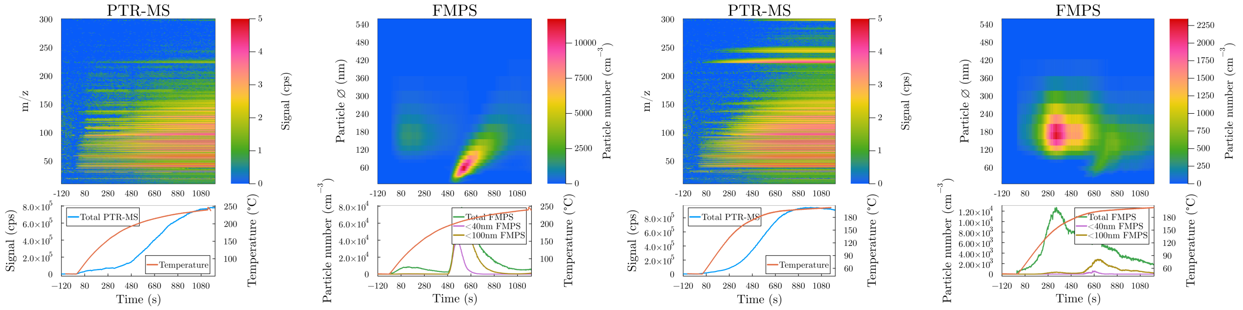

$c_p = 1.1\,\mathrm{MPa}, v = 4\,\mathrm{m\,s^{-1}}$

(w) test 18.1 (NAO) (x) test 18.2 (NAO) (y) test 18.3 (NAO)

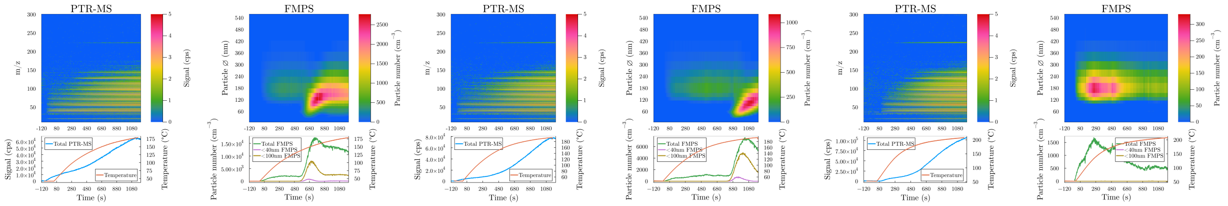

Figure S3: Time-resolved measurements. For each experiment: (top left) PTR-MS signals shown as fonction of mass-to-charge ratios (m/z) on a logarithmic heatmap; (top right) FMPS data displaying particle number concentration across different diameter ranges; (bottom left) integrated signals showing total PTR-MS response (blue); (bottom right) particle number concentrations in three size fractions ($< 40\,\mathrm{nm}$, $< 100\,\mathrm{nm}$, and total); with temperature profile (orange).

5 Onset Temperature

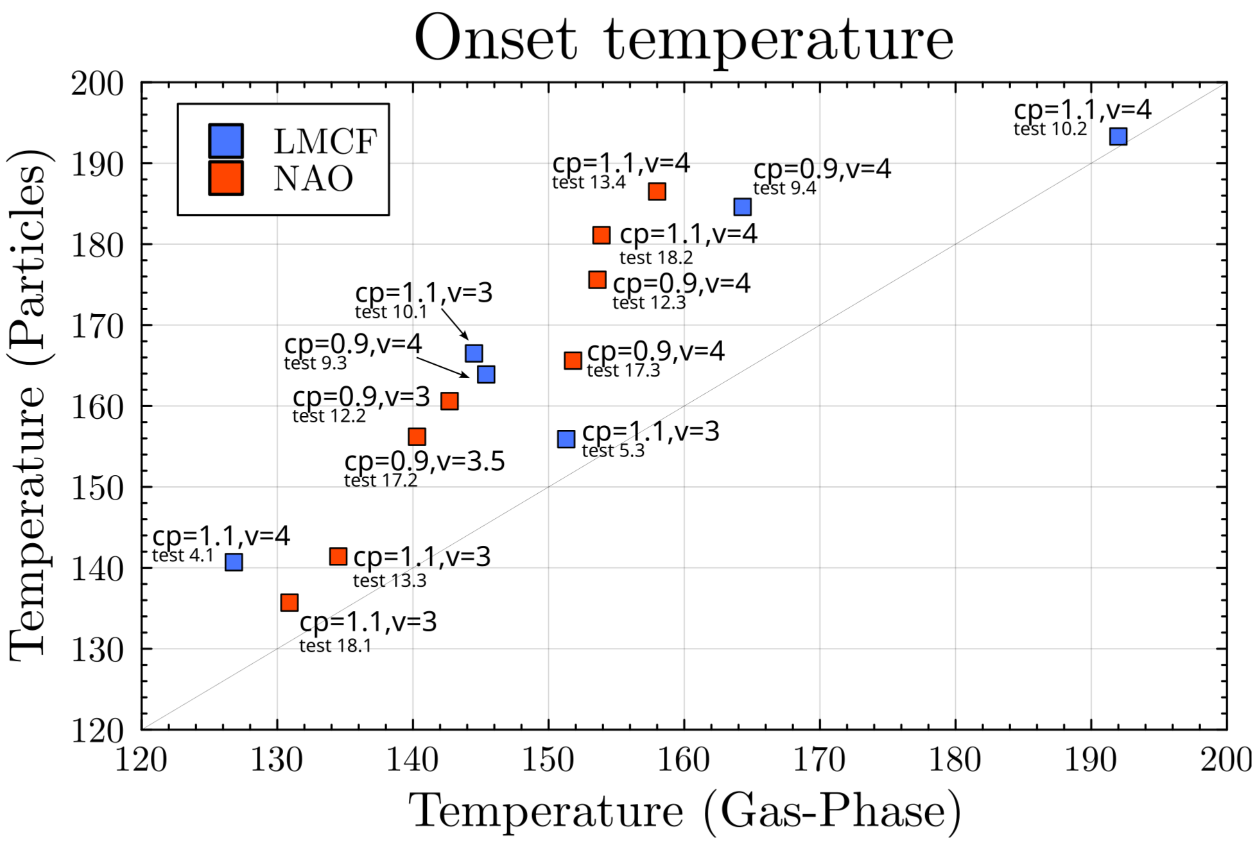

Figure S4: Onset temperature observed for particle formation versus gaseous emissions.

6 Peak Table

The methodology and results for identifying the VOCs are presented in this section.

6.1 Methodology

The list of peaks is generated from a characteristic mass spectrum. This mass spectrum has been chosen averaging over the entire acquisition time of the test 13.4 (NAO, $v = 4\,\mathrm{m\,s^{-1}}$, $c_p = 1.1\,\mathrm{MPa}$, see Table S2). For this averaging operation, individual spectra, recorded every second, are first of all rigorously mass-calibrated according to a function of the form:

$$m(t) = a\,t^c + b$$

where the calibration parameters a, b, c, are determined by taking the maximum intensity of 3 distinguishable peaks, corresponding to NH_3^+ (m/z 17.026), $C_5H_8O_2H^+$ (m/z 101.060) and $C_6H_4I_2H^+$ (m/z 330.848). This latest compound is an internal calibrant of the instrument.

All mass spectra signals are then averaged, interpolating beforehand each of them on an average mass axis to fit exactly every time the same x-axis. This method is computationally rather cumbersome but maximizes precision in peak calibration and the resulting spectrum resolution. The achieved uncertainty for a mass of m/z 150 is below 0.002, therefore allowing us to distinguish isotopes and provide a reliable chemical atomic composition of every peak.

Once an average mass spectrum has been calculated, each peak on these data is individually identified by taking its center on the maximum intensity of its respective Gaussian. A prominence criterion of 10 cps is applied to discriminate only peaks that appear distinctly and indisputably from each other in the mass spectrum. This operation results in a mass peak list of 210 entries.

This mass peak list is then compared with an in-house database containing the atomic composition of over 1000 molecules to assign each identified mass its closest molecular formula. The criterion to attribute a chemical formula to each mass is based on minimizing the

difference between the value of a mass in the spectrum list and that in the database. The list of the 210 molecules is thus assigned automatically to a chemical formula, and ultimately reviewed manually using Ionicon's PTR-Viewer software, and eventually slightly adjusted. The high resolution of the mass spectrometer and the precise calibration give us the confidence to deliver reliable results.

This technique is implemented with the Julia programming language and supplied in a package named *MassSpec.jl* under development. All this has been archived and provided on Zenodo, along with the raw data and the result of the processing.

6.2 List of VOCs

Table S5: Conditions: $v = 4\,\mathrm{m\,s^{-1}}$, $c_p = 1.1\,\mathrm{MPa}$, for both LMCF and NAO materials. Δ column is the signal background subtracted (absolute measurement in count per second). δ column is the signal background divided (measurement relative to the background). The list is sorted in alphabetical order.

		LMCF				NAO			
**mass**	**m/z**	**signal**	**background**	**Δ**	**δ (ratio)**	**signal**	**background**	**Δ**	**δ (ratio)**
$(H_2O)_2H^+$	37.028	1.89E+05	1.89E+05	-8.25E+01	1.00	1.66E+05	1.73E+05	-6.74E+03	0.96
$(H_2O[18])_2H^+$	39.033	2.43E+02	2.32E+02	1.07E+01	1.05	2.09E+02	2.13E+02	-4.34E+00	0.98
$(H_2O)_3H^+$	55.039	4.33E+03	4.02E+03	3.17E+02	1.08	3.17E+03	3.09E+03	7.23E+01	1.02
$C_{10}H_{14}H^+$	135.117	6.84E+02	4.80E+02	2.04E+02	1.43	9.36E+02	4.00E+02	5.36E+02	2.34
$C_{10}H_{16}H^+$	137.132	8.27E+02	3.25E+02	5.02E+02	2.55	1.01E+03	2.00E+02	8.12E+02	5.05
$C_{10}H_{16}OH^+$	153.127	2.07E+02	1.03E+02	1.04E+02	2.01	6.79E+02	1.02E+02	5.78E+02	6.69
$C_{10}H_{20}OH^+$	157.159	3.27E+02	2.45E+02	8.23E+01	1.34	2.42E+02	1.61E+02	8.13E+01	1.51
$C_{11}H_{18}H^+$	151.148	5.72E+02	2.65E+02	3.07E+02	2.16	6.46E+02	1.72E+02	4.74E+02	3.75
$C_2H_2{}^+$	26.015	3.49E+02	3.59E+01	3.13E+02	9.70	4.16E+02	3.07E+01	3.85E+02	13.55
$C_2H_2O^+$	42.01	2.09E+03	2.23E+02	1.87E+03	9.38	2.46E+03	1.99E+02	2.26E+03	12.32
$C_2H_2OH^+$	43.018	6.14E+04	6.03E+03	5.54E+04	10.19	6.66E+04	4.60E+03	6.20E+04	14.50
$C[13]CH_2OH^+$	44.005	1.78E+03	2.20E+02	1.56E+03	8.09	1.99E+03	1.78E+02	1.81E+03	11.21
$C_2H_2O[18]H^+$	45.008	6.89E+02	8.35E+01	6.06E+02	8.26	6.85E+02	8.48E+01	6.01E+02	8.08
$C_2H_3NH^+$	42.034	1.58E+04	1.78E+02	1.56E+04	88.69	1.68E+04	1.42E+02	1.66E+04	117.57
$C[13]CH_3NH^+$	43.054	5.45E+02	3.74E+01	5.07E+02	14.56	5.86E+02	2.75E+01	5.58E+02	21.30
$C_2H_3NOSiH^+$	86.006	3.71E+02	1.14E+01	3.60E+02	32.45	4.92E+02	8.39E+00	4.83E+02	58.61
$C_2H_3NSH^+$	74.006	1.73E+02	1.74E+01	1.56E+02	9.94	1.07E+03	1.79E+01	1.05E+03	59.94
$C_2H_4O_2{}^+$	60.021	5.11E+02	7.85E+01	4.33E+02	6.51	5.25E+02	6.47E+01	4.61E+02	8.12
$C_2H_4O_2H^+$	61.028	6.56E+04	6.88E+03	5.88E+04	9.54	5.92E+04	4.80E+03	5.44E+04	12.33
$C[13]CH_4O_2H^+$	62.032	1.87E+03	2.15E+02	1.66E+03	8.72	1.83E+03	1.63E+02	1.66E+03	11.21
$C_2H_4OH^+$	45.033	1.34E+05	3.65E+03	1.31E+05	36.83	1.30E+05	4.37E+03	1.26E+05	29.79
$C[13]CH_4OH^+$	46.037	4.49E+03	1.21E+03	3.28E+03	3.72	4.52E+03	1.24E+03	3.28E+03	3.63
$C_2H_5NH^+$	44.049	2.37E+02	5.69E+01	1.80E+02	4.17	3.58E+02	5.44E+01	3.04E+02	6.58
$C_2H_5NO_2H^+$	76.039	8.71E+02	7.58E+01	7.95E+02	11.49	1.15E+03	6.00E+01	1.09E+03	19.17
$C_2H_6O_2H^+$	63.044	3.96E+03	1.44E+02	3.82E+03	27.52	2.95E+03	1.42E+02	2.80E+03	20.72
$C_2H_6O_3H^+$	79.039	1.25E+03	1.64E+02	1.09E+03	7.63	9.94E+02	9.82E+01	8.96E+02	10.13
$C_2H_6OH^+$	47.049	1.12E+03	3.42E+02	7.81E+02	3.28	1.65E+03	3.91E+02	1.26E+03	4.22
$C_2H_8O_2SiH^+$	93.037	2.30E+03	1.88E+03	4.19E+02	1.22	2.88E+03	2.05E+03	8.27E+02	1.40

$C_2H_9NO_2Si_2H^+$	136.024	3.07E+02	2.19E+02	8.76E+01	1.40	3.91E+02	2.76E+02	1.15E+02	1.41
$C_3H_{11}NO_2SiH^+$	122.063	3.91E+02	8.63E+01	3.04E+02	4.53	4.49E+02	7.62E+01	3.73E+02	5.90
$C[13]C_2H_2H^+$	40.026	1.29E+03	1.70E+02	1.12E+03	7.62	1.36E+03	1.08E+02	1.25E+03	12.66
$C_3H_2N_3O_2H^+$	113.022	6.22E+02	3.47E+02	2.75E+02	1.79	1.07E+03	3.53E+02	7.16E+02	3.03
$C_3H_2O_2H^+$	71.013	5.20E+03	7.48E+02	4.45E+03	6.95	5.43E+03	5.05E+02	4.93E+03	10.76
$C_3H_2OH^+$	55.018	8.12E+02	1.55E+02	6.57E+02	5.25	9.96E+02	1.31E+02	8.64E+02	7.57
$C_3H_3{}^+$	39.023	4.21E+04	5.10E+03	3.70E+04	8.27	4.43E+04	3.29E+03	4.10E+04	13.46
$C_3H_3NH^+$	54.034	1.14E+03	2.51E+01	1.12E+03	45.56	1.08E+03	1.79E+01	1.06E+03	60.17
$C_3H_3NOH^+$	70.029	1.76E+03	2.60E+01	1.73E+03	67.50	1.67E+03	2.71E+01	1.64E+03	61.45
$C_3H_4H^+$	41.039	1.25E+04	1.59E+03	1.09E+04	7.89	1.28E+04	9.98E+02	1.18E+04	12.77
$C_3H_4N_3O_2H^+$	115.038	3.26E+02	9.74E+01	2.28E+02	3.34	3.33E+02	9.26E+01	2.41E+02	3.60
$C_3H_4O_2H^+$	73.028	7.23E+03	8.11E+02	6.42E+03	8.93	6.83E+03	6.08E+02	6.22E+03	11.22
$C[13]C_2H_4O_2H^+$	74.032	4.71E+02	8.58E+01	3.85E+02	5.49	4.94E+02	7.14E+01	4.23E+02	6.92
$C_3H_4O_3H^+$	89.023	1.09E+03	3.98E+02	6.92E+02	2.74	9.09E+02	2.84E+02	6.25E+02	3.20
$C_3H_4OH^+$	57.033	1.92E+04	8.57E+02	1.84E+04	22.41	1.58E+04	5.53E+02	1.53E+04	28.65
$C[13]C_2H_4OH^+$	58.037	1.07E+03	6.21E+01	1.01E+03	17.19	1.02E+03	4.56E+01	9.74E+02	22.36
$C_3H_5NH^+$	56.049	5.46E+02	2.11E+01	5.24E+02	25.83	5.97E+02	1.38E+01	5.83E+02	43.18
$C_3H_6H^+$	43.054	1.84E+03	3.60E+02	1.48E+03	5.12	2.17E+03	2.33E+02	1.94E+03	9.30
$C_3H_6N_3O_2H^+$	117.053	7.69E+02	1.71E+02	5.98E+02	4.51	9.10E+02	1.25E+02	7.84E+02	7.25
$C_3H_6O_2H^+$	75.044	5.82E+03	9.80E+02	4.84E+03	5.94	5.71E+03	6.61E+02	5.04E+03	8.63
$C_3H_6OH^+$	59.049	1.22E+05	1.49E+04	1.07E+05	8.16	1.08E+05	4.88E+03	1.03E+05	22.13
$C[13]C_2H_6OH^+$	60.053	5.53E+03	1.48E+03	4.05E+03	3.74	5.03E+03	1.06E+03	3.98E+03	4.76
$C_3H_8O_2H^+$	77.06	2.06E+03	2.47E+02	1.81E+03	8.32	1.42E+03	8.22E+01	1.34E+03	17.25
$C_3O_2H^+$	68.997	2.10E+02	3.47E+01	1.75E+02	6.05	1.95E+02	3.73E+01	1.57E+02	5.22
C_3OH^+	53.002	1.07E+04	1.40E+03	9.30E+03	7.66	1.27E+04	9.93E+02	1.17E+04	12.75
$C[13]C_2OH^+$	54.006	3.29E+02	4.36E+01	2.85E+02	7.54	3.79E+02	3.30E+01	3.46E+02	11.49
$C_4H_2H^+$	51.023	2.22E+02	4.42E+01	1.77E+02	5.01	3.13E+02	4.66E+01	2.66E+02	6.72
$C_4H_4H^+$	53.039	2.59E+03	2.47E+02	2.35E+03	10.50	2.92E+03	2.00E+02	2.72E+03	14.59
$C_4H_4O^+$	68.026	3.76E+02	1.35E+01	3.63E+02	27.83	3.85E+02	1.03E+01	3.75E+02	37.42
$C_4H_4O_2H^+$	85.028	4.20E+03	6.61E+02	3.54E+03	6.36	6.28E+03	5.56E+02	5.72E+03	11.29
$C[13]C_3H_4O_2H^+$	86.032	3.46E+02	5.06E+01	2.96E+02	6.85	4.42E+02	4.48E+01	3.98E+02	9.88
$C_4H_4O_3H^+$	101.023	6.35E+02	3.97E+02	2.39E+02	1.60	7.25E+02	4.03E+02	3.22E+02	1.80
$C_4H_4OH^+$	69.033	2.33E+03	1.25E+02	2.21E+03	18.70	2.26E+03	1.05E+02	2.16E+03	21.64
$C_4H_5NH^+$	68.049	9.45E+02	8.39E+01	8.61E+02	11.26	1.10E+03	6.30E+01	1.04E+03	17.49
$C_4H_6H^+$	55.054	1.80E+04	1.37E+03	1.66E+04	13.12	1.61E+04	9.33E+02	1.51E+04	17.21
$C[13]C_3H_6H^+$	56.058	1.77E+03	1.08E+02	1.66E+03	16.39	1.82E+03	7.50E+01	1.75E+03	24.28
$C_4H_6O_2H^+$	87.044	1.07E+04	1.34E+03	9.41E+03	8.03	1.27E+04	9.95E+02	1.17E+04	12.73
$C[13]C_3H_6O_2H^+$	88.047	7.40E+02	1.23E+02	6.17E+02	6.01	8.47E+02	9.85E+01	7.49E+02	8.60
$C_4H_6O_3H^+$	103.039	1.67E+03	7.46E+02	9.27E+02	2.24	1.90E+03	5.05E+02	1.40E+03	3.77
$C_4H_6OH^+$	71.049	9.69E+03	2.96E+02	9.40E+03	32.81	2.14E+04	2.32E+02	2.11E+04	92.18
$C[13]C_3H_6OH^+$	72.053	1.20E+03	8.98E+01	1.11E+03	13.40	1.92E+03	7.44E+01	1.84E+03	25.77
$C_4H_8H^+$	57.07	4.69E+03	1.24E+03	3.46E+03	3.80	3.96E+03	6.46E+02	3.31E+03	6.12
$C[13]C_3H_8H^+$	58.073	4.09E+02	8.27E+01	3.27E+02	4.95	4.17E+02	5.24E+01	3.64E+02	7.95
$C_4H_8O_2H^+$	89.06	2.17E+03	5.70E+02	1.60E+03	3.81	1.99E+03	4.23E+02	1.57E+03	4.72
$C_4H_8OH^+$	73.065	8.92E+03	4.55E+02	8.46E+03	19.61	1.20E+04	3.48E+02	1.16E+04	34.40
$C[13]C_3H_8OH^+$	74.068	1.04E+03	3.68E+02	6.74E+02	2.83	1.37E+03	3.19E+02	1.05E+03	4.29
$C_5H_{10}H^+$	71.086	7.12E+02	2.26E+02	4.86E+02	3.15	6.18E+02	1.27E+02	4.90E+02	4.85
$C_5H_{10}O_2H^+$	103.075	1.23E+03	4.10E+02	8.22E+02	3.00	1.04E+03	2.56E+02	7.88E+02	4.07
$C_5H_{10}OH^+$	87.08	2.81E+03	9.18E+01	2.72E+03	30.65	3.12E+03	6.28E+01	3.05E+03	49.59
$C_5H_4H^+$	65.039	6.25E+02	1.17E+02	5.08E+02	5.35	7.89E+02	9.45E+01	6.94E+02	8.34
$C_5H_4O_2H^+$	97.028	1.37E+04	3.52E+02	1.33E+04	38.86	1.24E+04	2.66E+02	1.21E+04	46.62
$C[13]C_4H_4O_2H^+$	98.032	1.30E+03	1.14E+02	1.18E+03	11.40	1.45E+03	9.71E+01	1.36E+03	14.97
$C_5H_4OH^+$	81.033	2.37E+03	1.56E+02	2.21E+03	15.20	3.04E+03	1.41E+02	2.90E+03	21.63
$C_5H_5NH^+$	80.049	1.46E+03	1.27E+02	1.33E+03	11.48	2.32E+03	1.04E+02	2.21E+03	22.29
$C_5H_5OH^+$	82.041	4.45E+02	2.08E+01	4.24E+02	21.36	8.28E+02	2.22E+01	8.05E+02	37.21
$C_5H_6{}^+$	66.046	2.27E+02	5.35E+01	1.73E+02	4.24	2.91E+02	4.65E+01	2.44E+02	6.26
$C_5H_6H^+$	67.054	4.75E+03	7.03E+02	4.05E+03	6.76	5.40E+03	5.36E+02	4.86E+03	10.08

$C_5H_6O_2H^+$	99.044	2.27E+03	3.62E+02	1.91E+03	6.27	3.98E+03	3.41E+02	3.63E+03	11.64
$C_5H_6OH^+$	83.049	8.46E+03	3.60E+02	8.10E+03	23.46	1.24E+04	2.68E+02	1.21E+04	46.11
$C_5H_7NO_2H^+$	114.055	6.31E+02	3.58E+02	2.72E+02	1.76	5.66E+02	2.08E+02	3.58E+02	2.72
$C_5H_8H^+$	69.07	8.66E+03	9.75E+02	7.69E+03	8.88	7.94E+03	6.59E+02	7.28E+03	12.05
$C[13]C_4H_8H^+$	70.073	9.77E+02	1.12E+02	8.65E+02	8.71	1.07E+03	7.12E+01	9.97E+02	15.00
$C_5H_8O_2H^+$	101.06	4.42E+03	1.65E+03	2.78E+03	2.68	5.60E+03	1.03E+03	4.57E+03	5.42
$C_5H_8OH^+$	85.065	1.17E+04	2.46E+02	1.14E+04	47.39	1.26E+04	1.98E+02	1.24E+04	63.58
$C[13]C_4H_8OH^+$	86.068	1.31E+03	1.42E+02	1.17E+03	9.23	1.45E+03	8.45E+01	1.36E+03	17.12
$C_5H_9NOH^+$	100.076	3.01E+03	1.90E+03	1.11E+03	1.59	1.85E+03	7.83E+02	1.07E+03	2.37
$C_6H_{10}H^+$	83.086	8.20E+03	9.58E+02	7.25E+03	8.57	7.20E+03	6.41E+02	6.56E+03	11.24
$C[13]C_5H_{10}H^+$	84.089	1.15E+03	7.86E+01	1.07E+03	14.63	1.30E+03	5.64E+01	1.25E+03	23.14
$C_6H_{10}O_2H^+$	115.075	2.07E+03	5.49E+02	1.52E+03	3.76	1.84E+03	4.10E+02	1.43E+03	4.49
$C_6H_{10}O_3H^+$	131.07	2.47E+03	1.17E+02	2.35E+03	21.05	3.14E+03	9.03E+01	3.05E+03	34.77
$C[13]C_5H_{10}O_3H^+$	132.074	1.96E+02	2.72E+01	1.69E+02	7.22	2.46E+02	2.20E+01	2.24E+02	11.17
$C_6H_{10}OH^+$	99.08	3.75E+03	1.93E+02	3.56E+03	19.48	4.49E+03	1.30E+02	4.36E+03	34.44
$C_6H_{12}O_2H^+$	117.091	7.34E+02	3.13E+02	4.20E+02	2.34	5.74E+02	2.22E+02	3.52E+02	2.59
$C_6H_{12}OH^+$	101.096	2.50E+03	1.21E+02	2.38E+03	20.68	2.46E+03	6.27E+01	2.40E+03	39.18
$C_6H_4O_2H^+$	109.028	5.57E+02	1.31E+02	4.26E+02	4.25	3.25E+03	1.72E+02	3.08E+03	18.88
$C_6H_5OH^+$	94.041	9.84E+02	7.26E+02	2.57E+02	1.35	1.41E+03	1.00E+03	4.13E+02	1.41
$C_6H_6{}^+$	78.046	7.55E+02	3.68E+01	7.18E+02	20.50	5.88E+02	5.85E+01	5.29E+02	10.05
$C_6H_6H^+$	79.054	5.10E+03	5.23E+02	4.58E+03	9.76	5.75E+03	3.49E+02	5.40E+03	16.50
$C_6H_6O_2H^+$	111.044	1.90E+03	8.12E+02	1.09E+03	2.34	4.02E+03	7.79E+02	3.24E+03	5.16
$C_6H_6OH^+$	95.049	5.27E+03	4.44E+03	8.27E+02	1.19	9.54E+03	5.69E+03	3.85E+03	1.68
$C[13]C_5H_6OH^+$	96.053	5.64E+02	3.05E+02	2.59E+02	1.85	9.59E+02	3.66E+02	5.93E+02	2.62
$C_6H_8H^+$	81.07	8.14E+03	1.77E+03	6.37E+03	4.59	9.56E+03	1.26E+03	8.31E+03	7.62
$C[13]C_5H_8H^+$	82.073	1.33E+03	1.89E+02	1.15E+03	7.07	1.57E+03	1.26E+02	1.44E+03	12.46
$C_6H_8O_2H^+$	113.06	8.35E+02	2.82E+02	5.53E+02	2.96	1.35E+03	2.73E+02	1.08E+03	4.94
$C_6H_8OH^+$	97.065	5.63E+03	2.56E+02	5.37E+03	21.99	8.60E+03	2.50E+02	8.35E+03	34.45
$C[13]C_5H_8OH^+$	98.068	9.13E+02	1.25E+02	7.88E+02	7.32	1.10E+03	7.99E+01	1.02E+03	13.80
$C_7H_{10}H^+$	95.086	7.04E+03	1.62E+03	5.42E+03	4.34	8.94E+03	1.14E+03	7.80E+03	7.86
$C[13]C_6H_{10}H^+$	96.089	9.76E+02	2.05E+02	7.70E+02	4.75	1.28E+03	1.48E+02	1.13E+03	8.67
$C_7H_{10}O_2H^+$	127.075	3.34E+02	1.07E+02	2.26E+02	3.11	6.31E+02	1.29E+02	5.02E+02	4.88
$C_7H_{10}OH^+$	111.08	2.47E+03	2.54E+02	2.21E+03	9.70	4.90E+03	2.94E+02	4.60E+03	16.65
$C_7H_{12}H^+$	97.101	7.75E+03	5.47E+02	7.20E+03	14.17	6.45E+03	3.32E+02	6.12E+03	19.44
$C[13]C_6H_{12}H^+$	98.105	8.46E+02	5.09E+01	7.96E+02	16.63	9.16E+02	3.43E+01	8.82E+02	26.74
$C_7H_{12}O_2H^+$	129.091	5.71E+02	2.05E+02	3.65E+02	2.78	7.01E+02	1.88E+02	5.13E+02	3.73
$C_7H_{12}OH^+$	113.096	2.11E+03	1.17E+02	1.99E+03	18.04	2.77E+03	1.06E+02	2.66E+03	26.20
$C_7H_{14}OH^+$	115.112	6.16E+03	1.11E+02	6.05E+03	55.31	5.25E+03	7.38E+01	5.17E+03	71.08
$C[13]C_6H_{14}OH^+$	116.115	5.74E+02	1.46E+01	5.60E+02	39.45	5.26E+02	1.09E+01	5.15E+02	48.16
$C_7H_4OH^+$	105.033	1.50E+03	2.46E+02	1.26E+03	6.12	2.20E+03	1.94E+02	2.00E+03	11.29
$C_7H_5NH^+$	104.049	3.18E+03	1.20E+02	3.06E+03	26.52	3.08E+03	4.95E+01	3.03E+03	62.24
$C_7H_5NOH^+$	120.044	1.53E+02	5.98E+01	9.30E+01	2.56	3.25E+02	4.06E+01	2.84E+02	7.99
$C_7H_6H^+$	91.054	1.40E+03	4.56E+02	9.40E+02	3.06	3.38E+03	3.17E+02	3.07E+03	10.68
$C_7H_6O_2H^+$	123.044	4.42E+02	2.93E+02	1.50E+02	1.51	1.78E+03	4.28E+02	1.35E+03	4.16
$C_7H_6OH^+$	107.049	4.10E+03	3.60E+02	3.74E+03	11.40	6.44E+03	2.09E+02	6.23E+03	30.79
$C[13]C_6H_6OH^+$	108.053	5.77E+02	1.77E+02	4.00E+02	3.27	7.68E+02	1.11E+02	6.57E+02	6.91
$C_7H_8{}^+$	92.062	2.88E+02	7.28E+01	2.15E+02	3.95	6.50E+02	4.46E+01	6.06E+02	14.57
$C_7H_8H^+$	93.07	1.46E+03	3.58E+02	1.10E+03	4.08	3.69E+03	2.63E+02	3.42E+03	14.01
$C[13]C_6H_8H^+$	94.073	2.72E+02	8.14E+01	1.91E+02	3.34	5.18E+02	7.54E+01	4.42E+02	6.86
$C_7H_8O_2H^+$	125.06	3.59E+02	1.03E+02	2.55E+02	3.48	8.93E+02	1.48E+02	7.45E+02	6.03
$C_7H_8OH^+$	109.065	1.29E+03	1.04E+03	2.43E+02	1.23	1.68E+03	5.85E+02	1.10E+03	2.88
$C_8H_{10}{}^+$	106.078	1.04E+02	2.84E+01	7.60E+01	3.68	4.49E+02	1.55E+01	4.34E+02	29.05
$C_8H_{10}H^+$	107.086	8.48E+02	2.23E+02	6.25E+02	3.81	3.31E+03	1.46E+02	3.17E+03	22.67
$C[13]C_7H_{10}H^+$	108.089	2.26E+02	8.23E+01	1.44E+02	2.75	4.61E+02	4.75E+01	4.13E+02	9.69
$C_8H_{10}OH^+$	123.08	4.94E+02	2.49E+02	2.45E+02	1.98	8.64E+02	1.81E+02	6.84E+02	4.78
$C_8H_{12}H^+$	109.101	3.80E+03	1.11E+03	2.69E+03	3.42	5.44E+03	6.99E+02	4.74E+03	7.78
$C[13]C_7H_{12}H^+$	110.105	5.15E+02	1.30E+02	3.84E+02	3.95	7.09E+02	8.24E+01	6.26E+02	8.61

$C_8H_{12}O_2H^+$	141.091	3.38E+02	9.53E+01	2.42E+02	3.54	5.30E+02	1.04E+02	4.26E+02	5.11
$C_8H_{12}OH^+$	125.096	1.61E+03	2.52E+02	1.36E+03	6.41	2.65E+03	2.55E+02	2.40E+03	10.41
$C_8H_{14}H^+$	111.117	2.46E+03	3.70E+02	2.09E+03	6.67	2.60E+03	2.24E+02	2.38E+03	11.64
$C_8H_{14}O_2H^+$	143.107	8.22E+02	2.95E+02	5.27E+02	2.79	9.43E+02	2.56E+02	6.87E+02	3.69
$C_8H_{14}OH^+$	127.112	3.26E+03	3.57E+02	2.90E+03	9.14	3.76E+03	2.32E+02	3.53E+03	16.17
$C[13]C_7H_{14}OH^+$	128.115	3.23E+02	4.04E+01	2.83E+02	8.01	3.80E+02	2.78E+01	3.52E+02	13.67
$C_8H_{15}NH^+$	126.128	1.12E+03	5.57E+01	1.07E+03	20.17	1.43E+03	3.45E+01	1.39E+03	41.35
$C_8H_{16}O_2H^+$	145.122	2.19E+02	5.66E+01	1.63E+02	3.87	3.04E+02	8.84E+01	2.16E+02	3.44
$C_8H_{16}OH^+$	129.127	4.56E+03	2.49E+02	4.31E+03	18.35	5.04E+03	1.41E+02	4.90E+03	35.75
$C[13]C_7H_{16}OH^+$	130.131	4.83E+02	2.53E+01	4.57E+02	19.10	5.78E+02	1.79E+01	5.60E+02	32.22
$C_8H_{18}O_2H^+$	147.138	2.10E+02	2.09E+01	1.89E+02	10.05	2.48E+02	1.53E+01	2.33E+02	16.19
$C_8H_6OH^+$	119.049	2.41E+02	8.66E+01	1.54E+02	2.78	5.62E+02	8.24E+01	4.79E+02	6.82
$C_8H_8H^+$	105.07	3.72E+02	9.39E+01	2.78E+02	3.96	6.39E+02	7.28E+01	5.66E+02	8.78
$C_8H_8OH^+$	121.065	6.39E+02	4.43E+02	1.96E+02	1.44	9.48E+02	4.03E+02	5.46E+02	2.36
$C_9H_{10}H^+$	119.086	3.27E+02	1.24E+02	2.03E+02	2.63	5.98E+02	1.01E+02	4.98E+02	5.93
$C_9H_{12}H^+$	121.101	3.20E+02	1.52E+02	1.68E+02	2.10	8.21E+02	1.01E+02	7.20E+02	8.15
$C_9H_{12}OH^+$	137.096	1.57E+02	8.24E+01	7.48E+01	1.91	3.22E+02	1.08E+02	2.14E+02	2.98
$C_9H_{14}H^+$	123.117	1.23E+03	6.45E+02	5.83E+02	1.90	1.72E+03	5.46E+02	1.17E+03	3.14
$C[13]C_8H_{14}H^+$	123.12	2.33E+02	9.03E+01	1.42E+02	2.58	3.18E+02	7.56E+01	2.42E+02	4.20
$C_9H_{14}OH^+$	139.112	1.00E+03	2.19E+02	7.83E+02	4.58	1.26E+03	2.03E+02	1.06E+03	6.22
$C_9H_{16}OH^+$	141.127	5.46E+02	2.90E+02	2.56E+02	1.88	7.41E+02	2.43E+02	4.98E+02	3.04
$C_9H_{18}OH^+$	143.143	6.61E+02	4.37E+02	2.24E+02	1.51	6.38E+02	3.25E+02	3.13E+02	1.96
$CH_2O_2H^+$	47.013	1.11E+03	4.53E+02	6.59E+02	2.46	1.03E+03	3.96E+02	6.36E+02	2.61
CH_2OH^+	31.018	4.39E+03	4.08E+02	3.98E+03	10.74	4.39E+03	2.32E+02	4.16E+03	18.89
$CH_3{}^+$	15.023	2.09E+02	3.14E+01	1.78E+02	6.66	2.43E+02	2.09E+01	2.22E+02	11.61
CH_3NH^+	30.034	1.95E+02	1.51E+02	4.46E+01	1.30	2.47E+02	1.86E+02	6.06E+01	1.33
$CH_3OH(H_2O)H^+$	51.044	9.71E+02	2.12E+02	7.59E+02	4.58	7.16E+02	6.68E+01	6.49E+02	10.71
CH_4OH^+	33.033	1.42E+04	2.86E+03	1.14E+04	4.98	1.35E+04	1.12E+03	1.24E+04	12.06
79 $C[13]H_4OH^+$	34.037	2.25E+02	7.59E+01	1.49E+02	2.96	2.22E+02	6.13E+01	1.60E+02	3.62
CH_4SH^+	49.011	4.30E+01	5.58E+00	3.75E+01	7.71	7.49E+02	5.36E+00	7.44E+02	139.73
$CH_6O_3SiH^+$	95.016	1.05E+03	4.26E+02	6.28E+02	2.47	1.31E+03	5.08E+02	8.05E+02	2.59
$CS_2{}^+$	75.944	2.74E+02	2.67E+00	2.71E+02	102.40	6.36E+02	1.99E+00	6.34E+02	318.93
H_3O^+	19.018	8.62E+05	8.62E+05	-4.54E+02	1.00	7.44E+05	7.51E+05	-7.27E+03	0.99
$H_3O[17]^+$	20.022	7.81E+02	7.76E+02	4.92E+00	1.01	8.54E+02	8.46E+02	8.67E+00	1.01
$H_3O[18]^+$	21.022	1.93E+03	1.92E+03	9.54E+00	1.00	2.11E+03	2.06E+03	4.68E+01	1.02
$HCNH^+$	28.018	1.64E+02	1.05E+01	1.54E+02	15.67	1.78E+02	1.03E+01	1.68E+02	17.28
$HONOH^+$	48.008	2.65E+02	2.63E+02	1.65E+00	1.01	3.17E+02	2.70E+02	4.73E+01	1.18
N_2H^+	29.013	6.14E+02	6.43E+02	-2.83E+01	0.96	7.17E+02	7.20E+02	-2.34E+00	1.00
$NH_3{}^+$	17.026	6.96E+02	6.72E+02	2.34E+01	1.03	9.30E+02	8.54E+02	7.62E+01	1.09
$NH_4{}^+$	18.034	3.47E+04	2.92E+04	5.49E+03	1.19	4.10E+04	3.50E+04	6.03E+03	1.17
NO^+	29.997	1.80E+05	1.87E+05	-7.31E+03	0.96	2.39E+05	2.40E+05	-6.26E+02	1.00
$N[15]O^+$	30.995	6.82E+02	6.97E+02	-1.52E+01	0.98	8.67E+02	8.82E+02	-1.49E+01	0.98
$NO_2{}^+$	45.992	4.11E+03	4.31E+03	-1.95E+02	0.95	5.28E+03	5.21E+03	6.45E+01	1.01
$O_2{}^+$	31.989	1.47E+05	1.60E+05	-1.34E+04	0.92	2.34E+05	2.43E+05	-9.17E+03	0.96
$O[17]_2{}^+$	32.994	1.22E+02	1.28E+02	-6.40E+00	0.95	1.80E+02	1.81E+02	-1.31E+00	0.99
$O[18]_2{}^+$	33.994	5.41E+02	5.92E+02	-5.07E+01	0.91	8.26E+02	8.83E+02	-5.62E+01	0.94
$SiO(H_2O)_2H^+$	81	1.78E+02	1.25E+02	5.33E+01	1.43	2.68E+02	1.54E+02	1.13E+02	1.73
$SiO(H_2O)H^+$	62.99	1.18E+02	3.29E+01	8.48E+01	3.58	3.07E+02	2.78E+01	2.79E+02	11.06
$SiOH^+$	44.979	1.67E+02	5.88E+01	1.08E+02	2.83	5.77E+02	5.96E+01	5.18E+02	9.70
$SiOH_3{}^+$	46.995	7.04E+01	2.69E+01	4.34E+01	2.61	3.59E+02	2.58E+01	3.33E+02	13.92
m102.06		4.52E+02	1.48E+02	3.05E+02	3.06	5.43E+02	1.14E+02	4.29E+02	4.76
m102.097		3.84E+02	8.66E+01	2.97E+02	4.43	4.37E+02	1.09E+02	3.29E+02	4.02
m112.047		3.20E+02	1.37E+02	1.83E+02	2.34	5.71E+02	1.18E+02	4.53E+02	4.85
m112.083		3.08E+02	2.99E+01	2.78E+02	10.31	5.38E+02	3.55E+01	5.02E+02	15.13
m112.118		4.52E+02	4.49E+01	4.07E+02	10.05	6.83E+02	2.82E+01	6.54E+02	24.18
m114.096		2.54E+02	3.59E+01	2.18E+02	7.08	3.27E+02	4.46E+01	2.83E+02	7.34
m144.142		5.55E+02	6.23E+01	4.92E+02	8.91	6.08E+02	4.24E+01	5.65E+02	14.33

m223.075	1.76E+02	9.27E+01	8.28E+01	1.89	1.88E+04	8.84E+01	1.87E+04	212.48
m224.075	4.18E+01	2.26E+01	1.91E+01	1.85	3.15E+03	2.20E+01	3.13E+03	142.99
m225.055	9.83E+01	5.69E+01	4.14E+01	1.73	9.27E+03	6.22E+01	9.21E+03	148.97
m226.057	1.89E+01	1.28E+01	6.02E+00	1.47	1.50E+03	1.41E+01	1.49E+03	106.68
m227.046	2.12E+01	1.36E+01	7.66E+00	1.56	1.32E+03	1.61E+01	1.30E+03	81.75
m241.083	5.30E+00	3.72E+00	1.58E+00	1.42	2.87E+02	4.13E+00	2.83E+02	69.55
m243.069	9.53E+00	7.75E+00	1.78E+00	1.23	3.19E+02	1.07E+01	3.09E+02	29.75
m297.09	2.39E+01	1.88E+01	5.05E+00	1.27	3.38E+02	3.97E+01	2.99E+02	8.52
m59.032	7.54E+02	2.71E+02	4.84E+02	2.79	7.18E+02	9.56E+01	6.23E+02	7.51
m84.051	1.95E+03	7.80E+01	1.88E+03	25.05	2.17E+03	6.39E+01	2.11E+03	33.96
siloxanes	4.64E+02	2.61E+02	2.03E+02	1.78	3.77E+04	3.05E+02	3.74E+04	123.69
sum	9.06E+05	1.27E+05	7.79E+05	7.16	1.01E+06	9.80E+04	9.13E+05	10.31

7 Classification

Ratio of the Figure 2 is computed for every groups as

$$R = \frac{\sum_i \delta_i \times S_i}{S_T}, \tag{1}$$

where i corresponds to any chemical molecule of a group, S_i is its absolute signal, $S_T = \sum_i S_i$ is the total signal of the group and δ_i is the signal/background ratio. Both δ_i and S_i, were taken form the list of VOCs computed beforehand.

References

(S1) Nozière, B.; Durif, O.; Dubus, E.; Kylington, S.; Emmer, Å.; Fache, F.; Piel, F.; Wisthaler, A. The reaction of organic peroxy radicals with unsaturated compounds controlled by a non-epoxide pathway under atmospheric conditions. Physical Chemistry Chemical Physics **2023**, Publisher: The Royal Society of Chemistry.

(S2) Durif, O.; Piel, F.; Wisthaler, A.; Nozière, B. Strong Uptake of Gas-Phase Organic Peroxy Radicals ($ROO^{\bullet}$) by Solid Surfaces Driven by Redox Reactions. JACS Au **2024**,
